# Gleanings in Science and Medicine

**Published:** 1893-06-24

**Authors:** 


### Gleanings in Science and Medicine.

### INTERESTING CURIOS.

An interesting collection of curios is now on the way out to the Chicago World's Fair, where it will be exhibited in the pavilion erected by the Rosbach Table Water Company. These curios consist of a collection of Roman pottery found in the grounds at the Rosbach Spring, near Homburg; and in excavating a short time since a very valuable find was made in the form of a flint knife, which has been submitted to Herr Jacobi, who is conducting the excavations for the German Government. According to his judgment, reinforced by the illustrations and description in the pre-historical work of Wosinsky, this flint knife belongs to the "First Stone Period," and in Herr Jacobi's opinion it must therefore be 8,000 years old, that is to say, of the period of 5,000 or 6,000 years B.C. The Rosbach Spring was well known to the Romans, as there still exists, kept in perfect repair, the four walls of the well or spring, identified as of the Roman period; and seven feet below the level of the soil there are distinct signs of an old Roman pavement. Among the other curiosities sent to Chicago are a collection of horse-shoes, which were found at the level of this pavement.

### CREMATION.

There can be little doubt that in England the general feeling is at present against cremation. The political question regarding the law relating to burials which, for so many years, caused so much bad feeling between the clergy of the Church of England and the Nonconformists, is now settled. But the medical and sanitary question still remains. In Germany there is to be fresh legislation for the prevention of epidemics; and a proposal is made in the Reichstag for making cremation optional throughout the Empire. In England cremation is, within certain limits, already optional, but there is a strong feeling, partly sentimental and partly religious, against it. The recent debate in the German Reichstag brought out the fact that, among all parties, there was a strong inclination to favour this mode of performing the last rites. What the balance of parties is to be in Germany, in the immediate future, is, of course, at present very uncertain, but there can be little doubt that a new law of a stringent character will be passed for the prevention of epidemics. Will cremation be made compulsory in cases of death from infectious diseases? In England public opinion is not nearly ripe for such a proposal.

